# Anti–USAG-1 therapy for tooth regeneration through enhanced BMP signaling

**DOI:** 10.1126/sciadv.abf1798

**Published:** 2021-02-12

**Authors:** A. Murashima-Suginami, H. Kiso, Y. Tokita, E. Mihara, Y. Nambu, R. Uozumi, Y. Tabata, K. Bessho, J. Takagi, M. Sugai, K. Takahashi

**Affiliations:** 1 Department of Oral and Maxillofacial Surgery, Graduate School of Medicine, Kyoto University, Kyoto, Japan.; 2 Department of Disease model, Institute for Developmental Research, Aichi Human Service Center, Kasugai, Aichi, Japan.; 3 Laboratory of Protein Synthesis and Expression, Institute for Protein Research, Osaka University, Osaka, Japan.; 4 Department of Molecular Genetics, Division of Medicine, Faculty of Medical Sciences, University of Fukui, Fukui, Japan.; 5 Department of Biomedical Statistics and Bioinformatics, Graduate School of Medicine, Kyoto University, Kyoto, Japan.; 6 Laboratory of Biomaterials, Institute for Frontier Life and Medical Sciences, Kyoto University, Kyoto, Japan.; 7 Life Science Innovation Center, University of Fukui, 23-3 Matsuoka Shimoaizuki, Eiheiji-cho, Yoshida-gun, Fukui 910-1193, Japan.

## Abstract

*Uterine sensitization–associated gene-1* (*USAG-1*) deficiency leads to enhanced bone morphogenetic protein (BMP) signaling, leading to supernumerary teeth formation. Furthermore, antibodies interfering with binding of USAG-1 to BMP, but not lipoprotein receptor–related protein 5/6 (LRP5/6), accelerate tooth development. Since USAG-1 inhibits Wnt and BMP signals, the essential factors for tooth development, via direct binding to BMP and Wnt coreceptor LRP5/6, we hypothesized that USAG-1 plays key regulatory roles in suppressing tooth development. However, the involvement of USAG-1 in various types of congenital tooth agenesis remains unknown. Here, we show that blocking USAG-1 function through *USAG-1* knockout or anti–USAG-1 antibody administration relieves congenital tooth agenesis caused by various genetic abnormalities in mice. Our results demonstrate that *USAG-1* controls the number of teeth by inhibiting development of potential tooth germs in wild-type or mutant mice missing teeth. Anti–USAG-1 antibody administration is, therefore, a promising approach for tooth regeneration therapy.

## INTRODUCTION

Like beaks, nails, horns, and several eccrine glands, teeth are ectodermal organs. Tooth morphogenesis is regulated by a signal transduction network involving interactions between the epithelium and mesenchyme (*1*–*3*). Interactions involving positive and negative loops among bone morphogenetic protein (BMP), fibroblast growth factors, Sonic hedgehog, and Wnt pathways regulate the morphogenesis of individual teeth (*1*, *4*). While the number of teeth is usually strictly controlled in individual species (*5*), it can increase or decrease congenitally in about 1% of individuals (*6*–*8*). Conditions of decreases and increases in the usual number of teeth are called tooth agenesis and supernumerary teeth, respectively. Analyses of mouse models have begun to clarify the genetic factors and molecular and pathological mechanisms underlying these conditions (*4*, *9*).

Investigations of single-gene knockout (KO) mice have demonstrated that loss of function of *Usag-1*, also referred to as *Sclerostin domain containing 1* (*SOSTDC1*), *ectodin*, or *Wnt modulator in surface ectoderm* (*WISE*), *CCAAT/enhancer-binding protein beta* (*CEBPB*), *Sprouty homolog 2* (*SPRY2*), *sprouty homolog 3* (*SPRY3*), or *Epiprofin* (*EPFN*), result in the production of supernumerary teeth (*10*–*14*). Results from these studies suggest that de novo tooth formation may be regulated by a single candidate gene. Supernumerary teeth may result from the rescue of arrested teeth germ (*10*, *15*); we have previously reported the transformation of the residual deciduous incisor into supernumerary teeth in *USAG-1*deficient mice (*10*). USAG-1 is a bifunctional protein that antagonizes BMP and Wnt, the two signaling molecules essential for tooth development (*4*, *9*). The importance of BMP in supernumerary tooth formation was demonstrated by transplantation of incisor explants supplemented with BMP7 in *USAG-1*^+/−^ mice, which induced the development of supernumerary teeth (*16*). Hence, the administration of candidate molecules can result in whole tooth formation in suitable conditions. Furthermore, it has been suggested that BMP signaling is essential for morphogenesis of extra teeth (*16*, *17*), while Wnt signaling is important for supernumerary tooth formation (*15*, *18*). However, it is unknown whether BMP or Wnt signaling is required for the determination of tooth number.

Tooth agenesis is the result of arrested tooth development. Several genes responsible for congenital tooth ageneses, such as *Msx1*, *Runx2*, *Ectodysplasin A* (*EDA*), or *Pax9* (*4*, *6*, *7*), have been identified primarily using KO mouse models (*19*–*24*). We previously reported that tooth development arrested in *Runx2*^−/−^ mice, a mouse model for congenital tooth agenesis (*24*), was rescued in *Runx2*^−/−^/*USAG-1*^−/−^ mice, a supernumerary mouse model (*25*). While a clear link between USAG-1 and rescue of congenital agenesis has been established, it remains unknown whether local inhibition of USAG-1 function is sufficient to rescue tooth development. Clinical applications of targeted molecular drugs based on antibody preparations for a variety of diseases, such as rheumatoid arthritis and cancer, are increasingly common (*26*, *27*). The genetic mechanisms of supernumerary tooth formation suggest that a targeted molecular therapy for tooth regeneration can be a viable therapeutic approach.

This investigation aimed to generate and use a monoclonal anti–USAG-1 antibody, rather than genetic inhibition, for the local arrest and recovery of tooth development. To this end, we also performed experiments to determine whether BMP or Wnt signaling is dominant during tooth development.

## RESULTS

### Tooth formation recovery using murine models

**Fig. 1:**
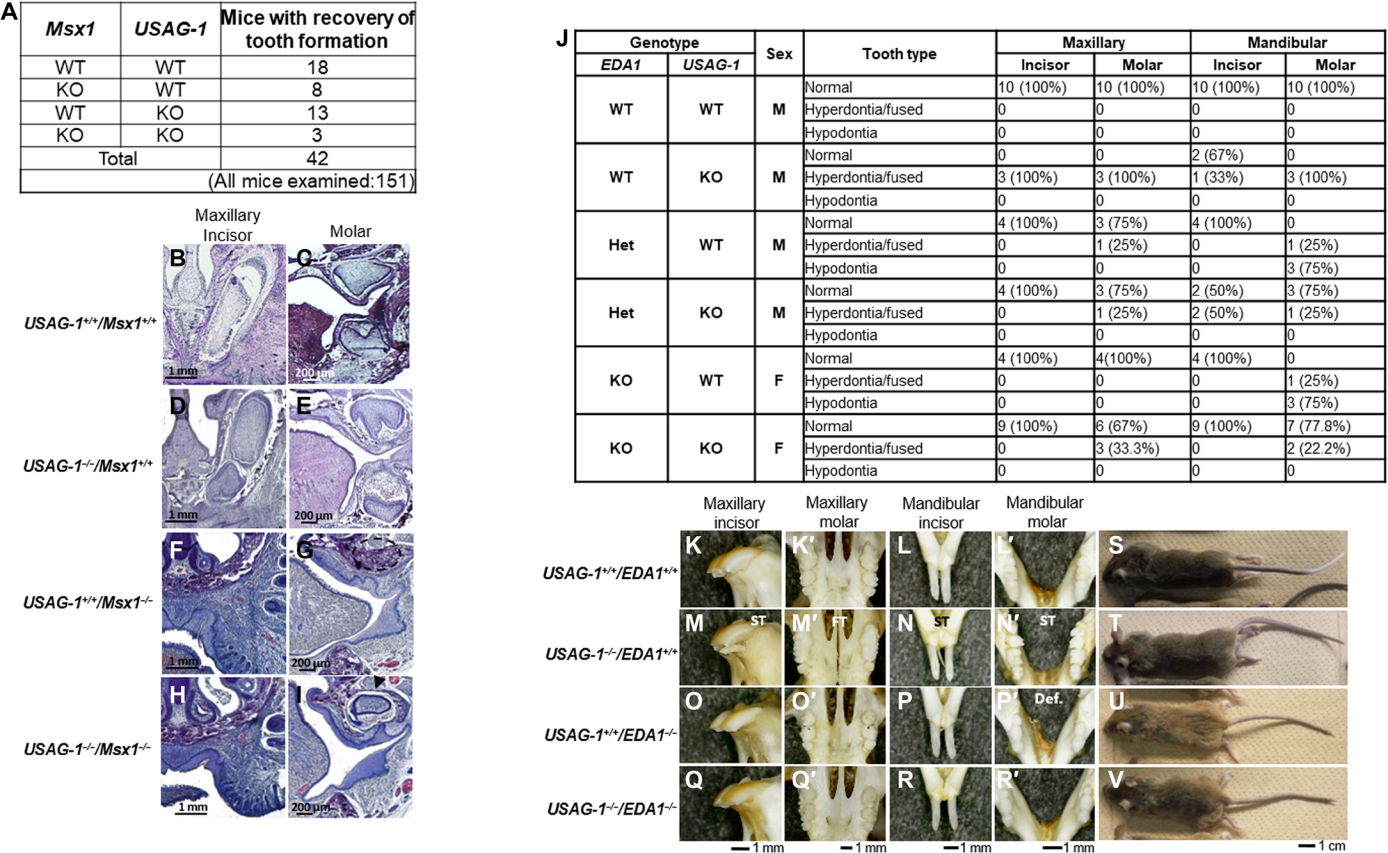
Recovery of tooth formation in double KO mice with congenital tooth agenesis and supernumerary teeth. (**A**) Number of mice with indicated genotypes. (**B** to **I**) Frontal hematoxylin and eosin–stained sections of the left maxillary incisor and third molar (M3) in *USAG-1*^−/−^*/Msx1*^−/−^ mice immediately after birth. (**J**) Summary of tooth phenotypes in 8-month-old F_2_ generation *EDA1/USAG-1* double-mutant mice. (**K** to **R′**) Representative tooth phenotypes in dry skulls of 8-month-old F_2_ generation *EDA1/USAG-1* double-mutant mice. (**S** to **V**) Ear hair, tail hair, and tail tip phenotypes. ST, supernumerary teeth; FT, fused teeth; Def., defect of teeth. Photo credit: H. Kiso, Kyoto University.

Phenotypic changes in an *Msx1*^−/−^*/USAG-1*^−/−^ mouse generated by mating mouse models of congenital tooth agenesis and supernumerary teeth were investigated. The development of both the maxilla and the mandible was arrested in the early stages. However, a cleft palate was additionally observed in *USAG-1^+/+^/Msx1*^−/−^ mice (Fig. 1, F and G). Although mouse offspring with a *USAG-1*^−/−^*/Msx1*^−/−^ background should have theoretically been obtained with one-sixteenth incidence, only 3 of 151 littermate mice had the *USAG-1*^−/−^*/Msx1*^−/−^ genotype (Fig. 1A). Histological evaluation revealed that all *USAG-1*^−/−^*/Msx1*^−/−^ mice had normal third maxillary molar teeth (Fig. 1, H and I).

Next, we analyzed *EDA1*^−/−^*/USAG-1*^−/−^ mice. As *EDA1* is located on the X chromosome, female *EDA1*^−/−^*/USAG-1*^−/−^ and male *EDA1^+/−^/USAG-1*^−/−^ mice are null for *USAG-1* and *EDA1*. These double KO mice had normal teeth, hyperdontia, or fused mandibular molars, whereas 75% of the female *USAG-1^+/+^/EDA1*^−/−^ and male *USAG-1^+/+^/EDA1^+/−^* mice had molar hypodontia in the mandible (Fig. 1, J to R′, and fig. S2). Hair loss behind the ear and tail kink, which are the typical phenotypes associated with tabby mice, were present in all *USAG-1* and *EDA1* double KO mice (Fig. 1V). These results suggest that *Usag-1*^−/−^ can rescue congenital tooth agenesis during early tooth development and promote morphogenesis of the whole tooth structure arrested in the late stage.

### Usag-1–neutralizing antibody recovers missing teeth and generates a whole tooth

**Fig. 2:**
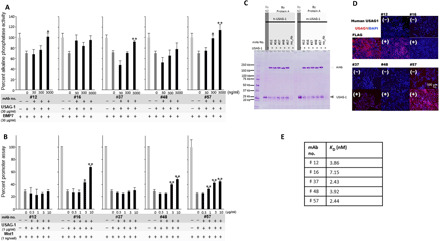
In vitro analyses of five types of USAG-1–neutralizing antibodies (#12, #16, #37, #48, and #57). (**A**) Neutralization of the antagonistic activity of BMP signaling by USAG-1 antibodies as assessed by alkaline phosphatase assay. (**B**) Neutralization of the antagonistic activity of Wnt signaling by USAG-1 antibodies in a Wnt reporter assay. (**C**) Binding between anti–USAG-1 antibody and human/mouse-PA-USAG-1 protein in pull-down assays. (**D**) Immunocytochemistry of human embryonic kidney (HEK) 293–expressing FLAG-tagged human USAG-1 protein. (**E**) *K*_D_ values of each USAG-1 antibody toward the mouse USAG-1 protein. mAb, monoclonal antibody; Ab, antibody; DAPI, 4′,6-diamidino-2-phenylindole.

To investigate whether inhibition of USAG-1 function rescues congenital tooth agenesis, we purified five mouse USAG-1 monoclonal antibodies (#12, #16, #37, #48, and #57) using a bioactive human USAG-1 recombinant protein derived from *Escherichia coli* as an antigen and *USAG-1*^−/−^ mice. USAG-1 is suggested to inhibit Wnt and BMP signals via direct binding to BMP and the Wnt coreceptor LRP5/6 (*28*, *29*). Therefore, these five antibodies were categorized into three different classes, based on their interfering abilities of the binding to both BMP and Wnt (#57), BMP (#12 and #37), or Wnt (#16 and #48) (Fig. 2, A and B). We confirmed that all antibodies could bind the mouse and human USAG-1 recombinant proteins (Fig. 2C), although #16 and #48 showed low affinity (Fig. 2, D and E). These results enabled the investigation of the function of USAG-1 with respect to BMP and Wnt signaling pathways for the determination of the number of teeth.

**Fig. 3:**
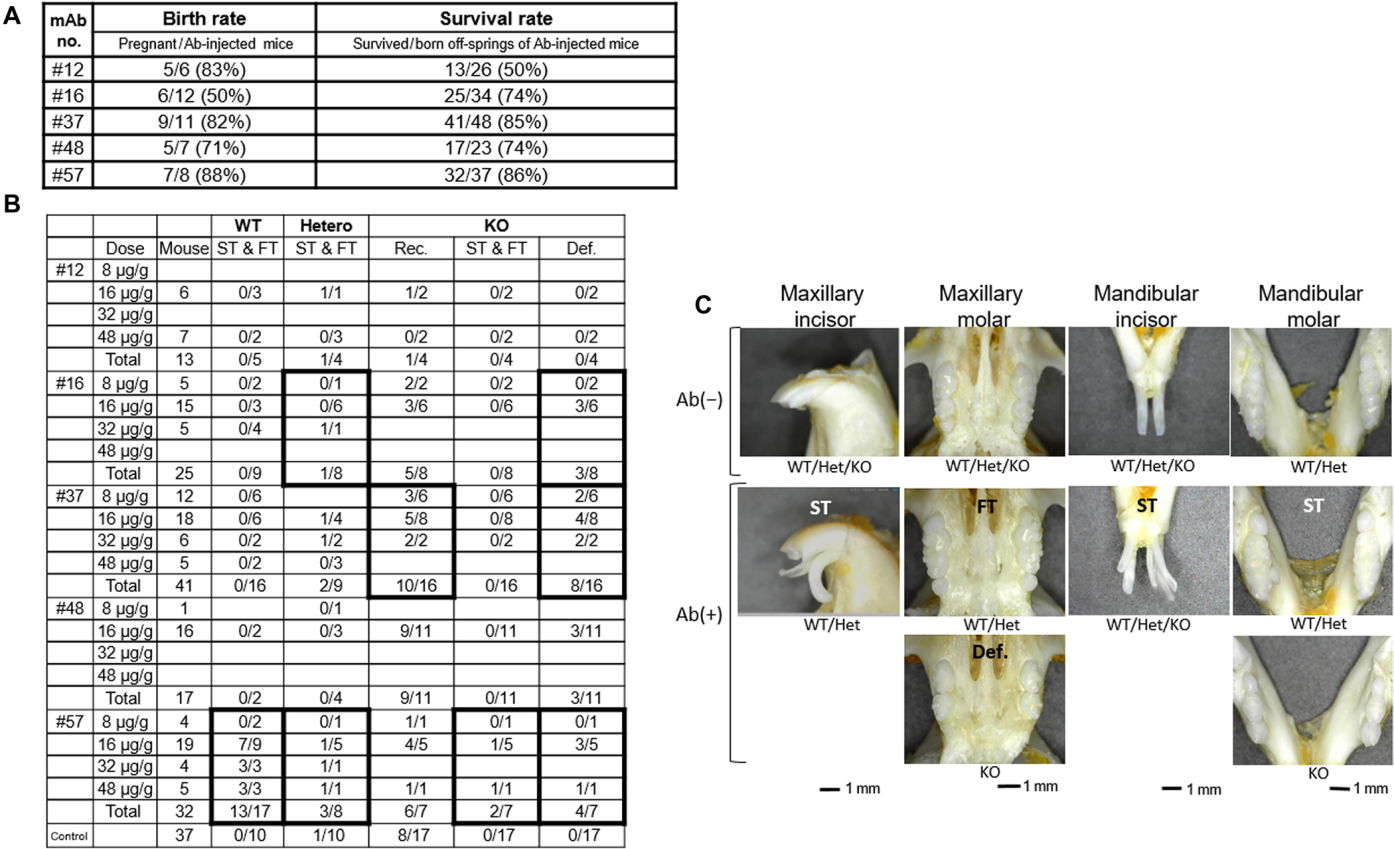
Recovery of tooth defects in *EDA1* mutant mice and whole tooth regeneration upon administration of USAG-1–neutralizing antibodies. (**A**) Offspring birth and survival rates. (**B**) Summary of incidence of tooth phenotypes, including supernumerary teeth and fused teeth (ST and FT), recovery of teeth (Rec.), and defect of teeth (Def.). (**C**) Representative tooth phenotype in dry skulls of 8-month-old mice. Photo credit: A. Murashima-Suginami, Kyoto University.

Each USAG-1–neutralizing antibody was systemically administered to *EDA1* pregnant mice. Low birth and survival rates were observed in mice administered USAG-1–neutralizing antibodies #12, #16, or #48 (Fig. 3A). USAG-1–neutralizing antibodies #16, #37, #48, and #57 rescued molar hypodontia in the mandible of *EDA1*^−/−^ mice compared with control mice (Fig. 3, B and C, and fig. S3). USAG-1–neutralizing antibody #37 reversed hypodontia at a high rate and in a dose-dependent manner (Fig. 3B). In addition, USAG-1–neutralizing antibodies #12, #16, #37, and #57 led to the production of supernumerary teeth in the maxillary incisor, mandibular incisor, or molar of *EDA1* KO/hetero mice (Fig. 3, B and C, and fig. S3). Unexpectedly, USAG-1–neutralizing antibody #57 induced the formation of supernumerary teeth in the maxillary incisor, mandibular incisor, or molar of wild-type mice at a high rate and a dose-dependent manner (Fig. 3, B and C, and fig. S3). However, fused molars were observed instead of supernumerary teeth in the maxillary molar region (Fig. 3C and fig. S3). Both antibodies neutralized BMP signaling antagonistic function, at least in vitro (Fig. 3, B and C, and fig. S3). These results indicate that BMP signaling is essential for determining the number of teeth in mice. Furthermore, a single systemic administration of a neutralizing antibody can generate a whole tooth.

### USAG-1–neutralizing activity generates a whole tooth by affecting BMP signaling

**Fig. 4:**
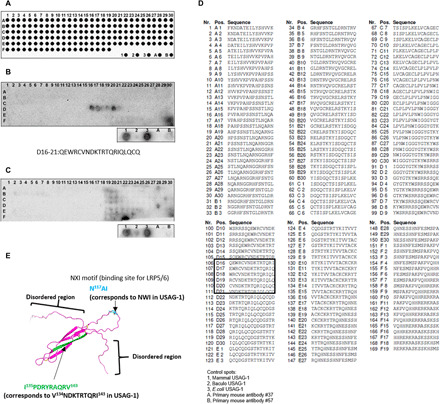
Epitope mapping of neutralizing USAG-1 antibodies #37 and #57. (**A**) The pattern of 14-mer peptide spots on the membrane from A1 to F19 [recombinant USAG-1 protein from: 1, mammalian cells; 2, Baculovirus; and 3, *E. coli*; USAG-1 antibodies (A) #37 and (B) #57]. (**B**) The peptide array probed with USAG-1 antibody #37. (**C**) The peptide array probed with USAG-1 antibody #57. (**D**) The number and sequence of the 14-mer peptide from A1 to F19. (**E**) Suggested 3D nuclear magnetic resonance structure model of mouse USAG-1 protein. Green, the epitope for USAG-1 antibody #37. Sky blue, the binding site for LRP5/6 (NX1 motif).

To determine the epitope of USAG-1–neutralizing antibodies #37 and #57, we performed epitope mapping using 169 linear peptides, including 20 sequential amino acids (Fig. 4, A and D). USAG-1–neutralizing antibody #37 specifically reacted with six overlapping peptides (D16-D21) spanning the region Q^129^EWRCVNDKTRTQRIQLQCQ^148^, suggesting that the epitope is localized within the central 10-residue segment containing the sequence VNDKTRTQRI (Fig. 4B). Although the three-dimensional (3D) structure of USAG-1 is unknown, its high sequence homology with sclerostin (SOST) that belongs to the same BMP antagonist DAN family enabled us to build a homology model of mouse USAG-1 using the nuclear magnetic resonance structure of SOST (Fig. 4E) (*28*). It was revealed that the epitope recognized by antibody #37 lies on the surface-exposed edge strand of the central β sheet of USAG-1, consistent with the ability of #37 to recognize native USAG-1. This region is located far from the NXI motif, which is the binding site for LRP5/6 (Fig. 4E) (*29*), suggesting that this antibody does not block USAG-1 interaction with the Wnt coreceptor LRP5/6. Antibody #37 did not affect the Wnt1-antagonizing activity of USAG-1 (Fig. 2B). In contrast to #37, antibody #57 did not show reactivity toward any of the USAG-1–derived overlapping peptides (Fig. 4C), indicating that it recognizes a 3D epitope present on the USAG-1 surface.

**Fig. 5:**
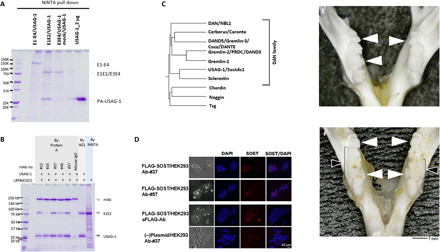
USAG-1–neutralizing antibodies sufficient for generating a whole tooth (#37 and #57) inhibit the antagonistic function of BMP but not Wnt signaling. (**A**) Interaction between the extracellular E1/E2 domain of LRP6 and mouse USAG-1 protein. (**B**) Blocking of the interaction between the extracellular domain of LRP6 E1/E2 and mouse USAG-1 protein by USAG-1 antibodies (#16). IgG, immunoglobulin G. (**C**) Dendrogram of DAN family proteins that are BMP antagonists. (**D**) Cross-reactivity of the USAG-1 antibody #57 to the SOST protein expressed in HEK293 cells. (**E**) Phenotype of the mandibular molar in the dry skull of a *USAG-1*^−/−^ mouse. (**F**) Phenotype of the mandibular molar in the dry skull of a *USAG-1*^−/−^ mouse administered a mix of USAG-1 antibodies (#12, #16, #37, #48, and #57). White arrowheads indicate supernumerary teeth; black arrowheads indicate enlarged fused teeth. Photo credit: A. Murashima-Suginami, Kyoto University.

It has been established that the endogenous Wnt pathway inhibitor SOST exerts its inhibitory effect by binding to the “E1” domain of Wnt coreceptor LRP6 (*30*). As described in the previous section, the conservation of the LRP6-binding motif NXI in USAG-1 strongly suggests that it binds to the same domain of LRP6 as well. We evaluated the USAG-1 binding to the human LRP6 ectodomain fragments of varying lengths. As shown in Fig. 5A, stoichiometric binding of USAG-1 was observed with the E1-E2 domain fragment of LRP6, confirming the prediction that the binding site was located in the E1 domain. In contrast, no binding was observed with E1-E4 or E3-E4 fragments. The lack of binding with E1-containing E1-E4 fragment can be explained by the fact that the NXI-binding surface of E1 is occluded in the context of the whole ectodomain of LRP6, which shows a highly curved “C-shape” in the electron microscopic images (*31*). We then investigated whether the USAG-1–neutralizing antibodies can interfere with the LRP6–USAG-1 interaction. As shown in Fig. 5B, near-complete inhibition was observed with antibody #16, while #48 exhibited partial inhibition. This finding was consistent with their ability to inhibit the Wnt-modulating activity of USAG-1 (Fig. 2B). Three other antibodies (#12, #37, and #57) did not affect the binding of USAG-1 to LRP6 E1-E2, corroborating their inability to counteract the Wnt-modulating capability of USAG-1 (Fig. 2B). On the basis of these results, we conclude that neutralizing the antagonizing effect of USAG-1 on BMP rather than Wnt signals is more effective in achieving substantial phenotypic changes in mice, i.e., recovering missing teeth or making a whole tooth.

To investigate the functional differences between antibodies #37 and #57 with respect to BMP signaling, we analyzed the cross-reactivity of these antibodies with members of the DAN subfamily (Fig. 5C). We detected a faint signal for SOST in transfected human embryonic kidney (HEK) 293 cells using immunohistochemistry with antibody #57 but not with #37 (Fig. 5D and fig. S4). This weak cross-reactivity with SOST is likely due to the similarities in the 3D structures of SOST and USAG-1 (*28*). Furthermore, systemic administration of an antibody mixture containing antibodies #12, #16, #37, #48, and #57 increased the number of supernumerary teeth and the size of fused teeth in the mandible of *USAG-1*^−/−^ mice (Fig. 5, E and F). These results suggest that antibody #57 may inhibit the genetic redundancy responsible for supernumerary tooth formation by affecting SOST, a BMP antagonist.

**Fig. 6:**
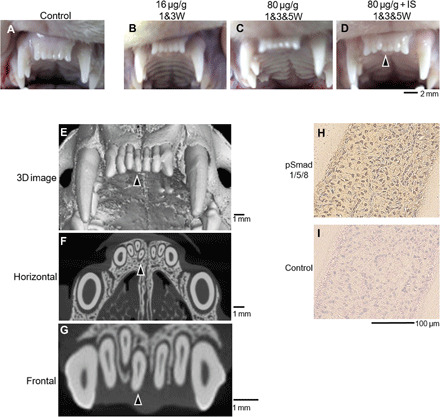
Supernumerary tooth of maxillary incisors of ferrets upon administration of USAG-1–neutralizing antibody #37. (**A** to **D**) Maxillary incisors of ferrets to different doses of administration USAG-1–neutralizing antibody #37. (**E** to **G**) Micro-computed tomography (micro-CT) image of Fig. 6D. (**H** and **I**) Immunolocalization of phosphorylated Smad1/5/8 (pSmad1/5/8) for supernumerary teeth. Arrowheads indicate supernumerary teeth. IS, immunosuppression. Photo credit: A. Murashima-Suginami, Kyoto University.

Last, to confirm that USAG-1–neutralizing activity affects BMP signaling to generate a whole tooth in a nonrodent model, we systemically administered antibody #37 to postnatal ferrets that had both deciduous and permanent teeth. We observed supernumerary tooth formation in maxillary incisor like the third dentition, although a five times higher concentration, three administrations of antibody #37, and immunosuppression were required (Fig. 6, A to D). The supernumerary tooth was likely to have a similar shape to the usual permanent incisor, located to the lingual side of permanent teeth, whereas a shorter root seemed to be growing (Fig. 6, E to G). Therefore, this supernumerary incisor might be categorized as the third dentition (*32*). Furthermore, phosphorylated Smad-positive cells were observed within pulp of supernumerary tooth (Fig. 6, H and I).

## DISCUSSION

Single systemic administration of USAG-1–neutralizing antibodies that interfere mainly with BMP signaling (#37 and #57) rescued tooth agenesis in *EDA1*-deficient mice and led to the efficient formation of a whole tooth in a dose-dependent manner in wild-type mice. To the best of our knowledge, the identification of targeted antibodies that can promote tooth regeneration has not been reported earlier. The antibodies generated in the present study neutralized the antagonistic action of USAG-1 on BMP signaling, and reduced LRP5/6 dosage rescued the *USAG-1*–null phenotype, including supernumerary tooth formation (*15*). However, Wnt signaling involvement cannot be excluded based on these findings because several mice were not born or did not survive. Thus, it is necessary to perform further experiments such as epitope binning involving higher numbers of USAG-1–neutralizing antibodies and detailed analyses of recombinant USAG-1 protein epitopes.

We observed links between several causative genes, including *Msx1* and *USAG-1*, with the recovery of congenital tooth agenesis but not cleft palate in *Msx1*-deficient mice (Fig. 1J). A single systemic administration of USAG-1–neutralizing antibodies targeting only the BMP signaling pathway rescued tooth agenesis in *EDA1* deficient mice but did not affect other phenotypes associated with this lineage. Conversely, *USAG-1* abrogation only rescued cleft palate development in *Pax9*-deficient mice, which modulated Wnt but not BMP signaling (*33*). Small-molecule Wnt agonists also corrected the cleft palate in *Pax9*-deficient mice (*34*). This indicates that the USAG-1–neutralizing antibody did not cure all tooth agenesis cases but that the mutations in causative genes for congenital tooth agenesis may constitute biomarkers for patient selection. Nevertheless, extensive studies are warranted for future clinical applications. EDA controls BMP activity (*35*), whereas EDAR acts on Wnt target genes (*36*, *37*). Congenital tooth agenesis may be rescued by administering a USAG-1–neutralizing antibody for BMP and not Wnt signaling. Furthermore, a single systemic administration of an EDA agonistic antibody in an *EDA*-deficient dog after birth rescued congenital tooth agenesis (*38*). Application of USAG-1–targeted neutralizing antibodies for tooth regeneration must be focused on congenital tooth agenesis with mutations of specific causative genes.

Further, we succeeded in obtaining USAG-1–neutralizing antibodies with the potential to generate a whole new tooth, even in wild-type mice. The phenotypic changes in these mice were similar to those in *USAG-1*-KO mice, suggesting that this antibody may rescue the rudimental tooth primordia in *USAG-1*–deficient mice. Human teeth, except for the permanent molars, are diphyodont (*32*). The first (deciduous) and second (permanent) generation of teeth are sometimes accompanied by a “third dentition” of rudimental teeth that can occur in addition to the permanent teeth (*32*). On the basis of an analysis of 78 patients with supernumerary teeth, we previously reported that the third dentition is a cause of supernumerary teeth in humans (*32*). Stimulation of the third dentition by targeted molecular therapy may be a viable approach for whole tooth regeneration. In the current study, we showed that systemic application of a USAG-1–neutralizing antibody could regenerate a whole tooth like the third dentition in ferrets, which are diphyodont animals with the similar dental pattern to human. However, the clinical application of this modality will require further investigation in nonrodent models, such as suncuses, dogs, or pigs, in addition to ferrets.

The development of a treatment method using cell-based tissue engineering is common in mainstream regenerative medicine. Although extensive research has been done in the field of tooth regeneration using tissue engineering techniques (*39*, *40*), none of the available therapies are clinically applicable due to cost and safety issues. Although it is considered necessary to generate a new original tooth germ, in our investigation, we observed the presence of rudimental tooth primordia. Therefore, we did not have to create new tooth primordia even in the wild-type animals. The growth of tooth primordia is inhibited by USAG-1. Besides, congenital tooth agenesis associated with various genetic abnormalities is caused by arrested tooth development. For this reason, the conventional tissue engineering approach is not suitable for tooth regeneration. Our study outcomes show that cell-free molecular therapy targeting USAG-1 is effective in the treatment of a wide range of congenital tooth agenesis and the induction of third dentition.

## MATERIALS AND METHODS

### Study design

This study’s main objectives included the generation and use of a monoclonal anti–USAG-1 antibody to locally arrest and recover tooth development in mice. We also performed experiments to determine whether BMP or Wnt signaling modulated tooth development. This study was approved by the Animal Research Committee of Kyoto University (reference number: Med Kyo 11518), KAC Co. Ltd. (reference number: 19-1103), and the Recombinant DNA Experiment Safety Committee of Kyoto University (reference number: 180211). Experiments were performed in accordance with approved guidelines. All experiments were repeated at least three times. Sample sizes were chosen empirically to ensure adequate statistical power. All valid measurements were included in our analysis. No outliers were excluded. Primary data are provided in the figures or the Supplementary Materials.

### Animals

*USAG-1*^−/−^ mice with a 106-bp deletion in exon 1 were produced using the CRISPR-Cas system with a C57BL/6J genetic background (fig. S1) (Macrogen Co. Ltd., Seoul, South Korea). Dental anomalies similar to those described in previous reports (*10*), including incisal supernumerary teeth, fused maxillary molars, and supernumerary mandibular molars, were observed in *USAG-1*^−/−^ mice. *EDA1*-deficient mice (Tabby6: C57BL/6J *A^w-J^*-*Eda^Ta-6J^*/J) were obtained from the Jackson Laboratory (JAX stock #000338). *Msx1*-deficient mice with a 129S4/SvJae genetic background were provided by the Mutant Mouse Resource and Research Centers (MMRRC stock #000068-UCD). We interbred heterozygous *USAG-1* and *Msx1* mice and analyzed the F_2_ generation. To eliminate the influence of the mouse background, only F2 progeny *USAG-1*^−/−^/*Msx1*^−/−^ mice were analyzed. Polymerase chain reaction was performed using KOD FX NEO polymerase (KFX-201; TOYOBO, Osaka, Japan) and specific primers. Embryos were obtained by timed mating; day E0 started from midnight, before finding a vaginal plug. Outbred pregnant ferrets were purchased from Marshall BioResources Japan Co. Ltd. A subgroup of the offspring was maintained in immunosuppressive condition, as previously reported (*41*).

### Plasmid and recombinant proteins

Preparation of PA-tagged mouse USAG-1 recombinant protein from mammalian cells was performed as previously reported (*42*). Other tagged USAG-1 recombinant proteins, derived from *E. coli* or baculoviral expression systems (R&D systems Inc., MN, USA; MyBiosource, CA, USA), were used for the production of antibodies, as antigens, and in the solid phase and/or sandwich enzyme-linked immunosorbent assay (ELISA). Preparation of the E1-E4 domain of LRP6 was performed as previously reported (*30*). Expression vectors for mouse DAN family proteins were purchased from OriGene Technologies Inc. (Rockville, MD, USA).

### Generation and purification of anti–USAG-1 monoclonal antibodies

The anti–USAG-1 monoclonal antibodies were generated by ITM Co. Ltd. (Matsumoto, Japan) as previously described (*43*). Briefly, *USAG-1*^−/−^ mice were immunized with recombinant human USAG-1 protein. Two weeks later, the lymphocytes obtained from iliac lymph nodes were fused with SP2/0 mouse myeloma cells in the presence of 50% polyethylene glycol solution and were selected for 1 week on GIT medium (Wako Pure Chemical Corporation, Osaka, Japan) containing HAT as a supplement. The resultant hybridomas were screened by ELISA, and those secreting anti–USAG-1 monoclonal antibodies were identified. The culture supernatant (10 ml) was loaded onto a Protein G column (GE Healthcare, Chicago, IL, USA), and the antibody was adsorbed onto the column. Bound antibody was eluted using the elution buffer from the MAbTrap Kit (GE Healthcare). The eluted antibody was loaded on a centrifugal filter (Amicon Ultra-15; Millipore, Burlington, MA, USA) for buffer exchange with phosphate-buffered saline (PBS), and concentration was determined. Antibodies were stored at −80°C until use.

### Alkaline phosphatase assay

For determination of alkaline phosphatase (ALP) activity, C2C12 cells were seeded at a density of 6 × 10^4^ cells per well in 96-well plates. After the cells reached confluency, they were cultured in Dulbecco’s modified Eagle’s medium (DMEM; Sigma-Aldrich, St. Louis, MO, USA) supplemented with 15% fetal bovine serum (FBS), penicillin G (100 U/ml), streptomycin (100 μg/ml), recombinant mouse BMP7 protein (30 ng/ml or 300 ng/ml) (R&D systems), and recombinant mouse USAG-1 protein (0 to 3000 ng/ml) for 48 hours. Cells were washed twice with PBS and scraped in 0.05% Triton X-100. The cell suspension was sonicated on ice. Aliquots of supernatants were assayed for protein concentration and ALP activity (LabAssay ALP, FUJIFILM Wako Pure Chemical Corporation) as described.

### Luciferase reporter assay

To assess the neutralizing effects of the anti–USAG-1 antibodies on Wnt/β-catenin signaling modulated by recombinant mouse USAG-1, we used the TOP reporter system based on the dual-luciferase reporter assay system (Promega, Madison, WI, USA). Briefly, HEK293 cells (1.0 × 10^4^ cells per well in a 48-well plate) were transiently transfected with constitutively active herpes simplex virus thymidine kinase promoter-driven *Renilla* luciferase (20 ng per well) as an internal control, a β-catenin–responsive firefly luciferase reporter plasmid TopFlash (50 ng per well) (Millipore), and Wnt1 expression plasmid (1 ng per well) using Lipofectamine 3000 (Thermo Fisher Scientific, Waltham, MA, USA). After 4-hour incubation, the plasmids and the transfection reagent in DMEM supplemented with 10% FBS were replaced with a fresh medium containing recombinant mouse USAG-1 protein (1 μg/ml). Cells were harvested after 20 to 24 hours, and both firefly and *Renilla* luciferase activity were measured in duplicate or triplicate according to the manufacturer’s instructions. The firefly luciferase activity was normalized against the *Renilla* luciferase activity.

### Epitope mapping

Epitope mapping was performed by Kinexus Co Ltd. (Vancouver, Canada). Briefly, SPOT synthesis of two copies of a peptide array (15-mer peptide scan of a protein with 183 amino acids; human Sostdc1 without signal peptide) was performed on a cellulose membrane. Two of the synthesized copies of the peptide array were incubated with primary mouse USAG-1 antibodies (0.3 g/ml), and the bound antibody was detected by incubating the arrays with the detection reagent (1:25,000 dilution; HRPalpaca anti-mouse antibody) and subsequent treatment with electrochemiluminescence reagent.

### Immunoprecipitation

Reactivity of each monoclonal antibody (mAb) with native USAG-1 in solution was evaluated by immunoprecipitation. Briefly, 5 μg of purified anti–USAG-1 mAbs was incubated with 15 μl of Protein A-Sepharose (GE Healthcare) for 2.5 hours at 15° to 25°C, followed by a brief wash with PBS. The beads were incubated with the culture supernatants of the Expi293F cells transiently transfected with either mouse or human *USAG-1* containing N-terminal PA tag (*42*). After extensive washing with PBS, the bound proteins were eluted from the beads by adding SDS sample buffer and then analyzed by SDS–polyacrylamide gel electrophoresis (PAGE) using 5 to 20% gradient gel under nonreducing conditions.

### Bio-layer interferometry

Binding kinetics of anti–USAG-1 antibodies were analyzed using bio-layer interferometry with Octet RED system (ForteBio, Fremont, CA, USA). Binding assays were performed in 96-well microtiter plates at 25°C with orbital sensor agitation at 1000 rpm. Amine reactive (AR2G) sensors were immobilized with each antibody dissolved at 10 to 20 μg/ml in 10 mM sodium acetate buffer (pH 6.0) followed by quenching with 1 M ethanolamine (pH 8.5). Purified mouse USAG-1 was serially diluted in a running buffer [20 mM Hepes and 150 mM NaCl (pH 7.2) containing 0.005% Tween 20] and added to different wells (final volume: 200 μl). The binding was monitored by dipping the sensors into the wells for 120 s, followed by dissociation in the running buffer for 120 s. After each binding experiment cycle, antibody-immobilized biosensors were regenerated by dipping in a regeneration buffer [10 mM glycine-HCl (pH 3.0)]. The *K*_D_ values were determined using Octet Data Analysis Software 7.1 (ForteBio) using a 1:1 global fitting model.

### LRP6-binding assay

Binding between USAG-1 and LRP6 ectodomain was evaluated as follows. The soluble human LRP6 ectodomain fragments containing different regions (E1-E4, residues 1 to 1244; E1-E2, residues 1 to 629; E3-E4, residues 630 to 1244) were C-terminally His-tagged and transiently expressed in Expi293F cells as described previously (*44*). After immobilizing onto Ni-NTA beads, they were further incubated with the culture supernatants of the Expi293F cells stably expressing mouse USAG-1 established previously (*42*). The bound USAG-1 was eluted together with the LRP6 fragments by SDS and analyzed by nonreducing SDS-PAGE. For the assessment of the ability of anti–USAG-1 antibodies to compete with LRP6 binding, Protein A beads were incubated with each antibody (step 1), followed by incubation with USAG-1 (step 2), and lastly with LRP6 E1-E2 fragment (step 3) to allow the formation of a ternary complex. The bound proteins were analyzed by nonreducing SDS-PAGE. The diminished intensity of the signal corresponding to the LRP6 E1-E2 fragment indicated the overlap of the binding sites for the antibody and LRP6.

### Analysis of teeth phenotypes

Pregnant *EDA1* mice at E13 of gestation (4 to 6 weeks) were intraperitoneally injected with anti–USAG-1 antibodies (16 μg/g). Offspring were analyzed at 5 weeks of age. After removing the skin, dissected maxillae and mandibles from the heads of the offspring were soaked in 0.02% proteinase K prepared in PBS at 37°C for 4 days and cleaned with 5% H_2_O_2_ at 15° to 25°C for 5 min. Last, they were rinsed in H_2_O and air-dried. Neonates were fixed in 4% paraformaldehyde and embedded in paraffin. Sections (7 mm) were cut and stained with hematoxylin and eosin. Offspring of ferrets at 1 and 3 weeks after birth or 1, 3, and 5 weeks were intraperitoneally injected with anti–USAG-1 antibodies (16 or 80 μg/g). They were analyzed by taking photographs and micro-computed tomography (micro-CT).

### Micro-CT analysis

We performed 3D micro-CT scans (inspeXio SMX-100CT; Shimadzu, Kyoto, Japan) on the maxillary incisors of ferrets, 13 weeks after birth. We converted CB files [512 × 512 pixels, 8 bits; voxel size, *x*:*y*:*z* = 1:1:1 (~0.06 mm per side)] to TIFF files, and 3D images were reconstructed and analyzed using computer imaging software (VGSTUDIO MAX; Volume Graphics GmbH., Heidelberg, Germany).

### Immunocytochemistry

Immunocytochemistry was performed using standard techniques. Briefly, HEK293 cells were seeded on poly-l-lysine–coated coverslips (Matsunami Glass Ind. Ltd., Osaka, Japan). FLAG-tagged DAN family protein expression plasmids were transfected (1 μg per well) into the cells using Lipofectamine 3000. After transfection (24 hours), the cells were fixed with 4% paraformaldehyde/PBS (Sigma-Aldrich) for 30 min. Next, the cells were washed with PBS three times and incubated in blocking buffer (10% bovine serum albumin/PBS) for 1 hour, followed by incubation in the mouse monoclonal anti–USAG-1 antibody or anti-FLAG antibody (4 ng/ml) (Sigma-Aldrich) in the blocking buffer overnight at 4°C. To visualize the immunoreactivity, the cells were incubated with Cy3-labeled secondary antibody (Jackson ImmunoResearch Laboratories, West Grove, PA, USA)/PBS (1:400) after being washed three times with PBS. Nuclear staining was performed using 4′,6-diamidino-2-phenylindole (Thermo Fisher Scientific).

### Immunohistochemistry

Paraffin-embedded sections of ferret was immunostained with primary rabbit polyclonal antibodies against phosphorylated Smad 1/5/8 (1:50; Merck KGaA, Darmstadt, Germany) and secondary biotinylated anti-rabbit/mouse antibodies (Nichirei Bioscience, Tokyo, Japan), as previously described (*11*, *32*). Sections were then counter-stained with hematoxylin, dehydrated in a graded series of ethanol and xylene, and covered with coverslips.

### Statistical analysis

Data are shown as means ± SEs. For comparing multiple conditions, a one-way analysis of variance (ANOVA) was performed, followed by two-tailed Dunnett’s multiple comparisons test. Statistical significance of differences was assessed as follows: *P < 0.05, **P < 0.01. Statistical analyses were performed using the SAS statistical software, version 9.4 (SAS Institute, Cary, NC).

## SUPPLEMENTARY MATERIALS

Supplementary material for this article is available at http://advances.sciencemag.org/cgi/content/full/7/7/eabf1798/DC1

## References

[1] J. Pispa, I. Thesleff, Mechanisms of ectodermal organogenesis. Dev. Biol. 262, 195–205 (2003). 14550785 10.1016/s0012-1606(03)00325-7

[2] Y.-Y. Tai, R.-S. Chen, Y. Lin, T.-Y. Ling, M.-H. Chen, FGF-9 accelerates epithelial invagination for ectodermal organogenesis in real time bioengineered organ manipulation. Cell Commun. Signal 10, 34 (2012). 23176204 10.1186/1478-811X-10-34 PMC3515343

[3] M. Hirayama, M. Oshima, T. Tsuji, Development and prospects of organ replacement regenerative therapy. Cornea 32 (Suppl. 1), S13–S21 (2013). 24104927 10.1097/ICO.0b013e3182a18e6c

[4] I. Thesleff, The genetic basis of tooth development and dental defects. Am. J. Med. Genet. A 140, 2530–2535 (2006). 16838332 10.1002/ajmg.a.31360

[5] R. H. Meadow, Teeth. Simon Hillson. Am. Anthropol. 89, 773–774 (1987).

[6] M. Yu, S.-W. Wong, D. Han, T. Cai, Genetic analysis: Wnt and other pathways in nonsyndromic tooth agenesis. Oral Dis. 25, 646–651 (2019). 29969831 10.1111/odi.12931 PMC6318069

[7] G. Galluccio, M. Castellano, C. La Monaca, Genetic basis of non-syndromic anomalies of human tooth number. Arch. Oral Biol. 57, 918–930 (2012). 22325622 10.1016/j.archoralbio.2012.01.005

[8] J. Machida, T. Nishiyama, H. Kishino, S. Yamaguchi, M. Kimura, A. Shibata, T. Tatematsu, M. Kamamoto, K. Yamamoto, S. Makino, H. Miyachi, K. Shimozato, Y. Tokita, Genetic epidemiology of tooth agenesis in Japan: A population- and family-based study. Clin. Genet. 88, 167–171 (2015). 25041097 10.1111/cge.12456

[9] K. Takahashi, H. Kiso, A. Murashima-Suginami, Y. Tokita, M. Sugai, Y. Tabata, K. Bessho, Development of tooth regenerative medicine strategies by controlling the number of teeth using targeted molecular therapy. Inflamm. Regen. 40, 21 (2020). 32922570 10.1186/s41232-020-00130-x PMC7461317

[10] A. Murashima-Suginami, K. Takahashi, T. Kawabata, T. Sakata, H. Tsukamoto, M. Sugai, M. Yanagita, A. Shimizu, T. Sakurai, H. C. Slavkin, K. Bessho, Rudiment incisors survive and erupt as supernumerary teeth as a result of USAG-1 abrogation. Biochem. Bioph. Res. Commun. 359, 549–555 (2007). 10.1016/j.bbrc.2007.05.148 17555714

[11] A. Murashima-Suginami, K. Takahashi, T. Sakata, H. Tsukamoto, M. Sugai, M. Yanagita, A. Shimizu, T. Sakurai, H. C. Slavkin, K. Bessho, Enhanced BMP signaling results in supernumerary tooth formation in USAG-1 deficient mouse. Biochem. Biophys. Res. Commun. 369, 1012–1016 (2008). 18329379 10.1016/j.bbrc.2008.02.135

[12] K. Saito, K. Takahashi, B. Huang, M. Asahara, H. Kiso, Y. Togo, H. Tsukamoto, S. Mishima, M. Nagata, M. Iida, Y. Tokita, M. Asai, A. Shimizu, T. Komori, H. Harada, M. MacDougall, M. Sugai, K. Bessho, Loss of stemness, EMT, and supernumerary tooth formation in Cebpb^−/-^Runx2^+/−^ murine incisors. Sci. Rep. 8, 5169 (2018). 29581460 10.1038/s41598-018-23515-y PMC5980103

[13] O. D. Klein, G. Minowada, R. Peterkova, A. Kangas, B. D. Yu, H. Lesot, M. Peterka, J. Jernvall, G. R. Martin, Sprouty genes control diastema tooth development via bidirectional antagonism of epithelial-mesenchymal FGF signaling. Dev. Cell 11, 181–190 (2006). 16890158 10.1016/j.devcel.2006.05.014 PMC2847684

[14] T. Nakamura, S. de Vega, S. Fukumoto, L. Jimenez, F. Unda, Y. Yamada, Transcription factor epiprofin is essential for tooth morphogenesis by regulating epithelial cell fate and tooth number. J. Biol. Chem. 283, 4825–4833 (2008). 18156176 10.1074/jbc.M708388200

[15] Y. Ahn, B. W. Sanderson, O. D. Klein, R. Krumlauf, Inhibition of Wnt signaling by Wise (Sostdc1) and negative feedback from Shh controls tooth number and patterning. Development 137, 3221–3231 (2010). 20724449 10.1242/dev.054668 PMC6512258

[16] H. Kiso, K. Takahashi, K. Saito, Y. Togo, H. Tsukamoto, B. Huang, M. Sugai, A. Shimizu, Y. Tabata, A. N. Economides, H. C. Slavkin, K. Bessho, Interactions between BMP-7 and USAG-1 (uterine sensitization-associated gene-1) regulate supernumerary organ formations. PLOS ONE 9, e96938 (2014). 24816837 10.1371/journal.pone.0096938 PMC4016158

[17] Y. Kassai, P. Munne, Y. Hotta, E. Penttilä, K. Kavanagh, N. Ohbayashi, S. Takada, I. Thesleff, J. Jernvall, N. Itoh, Regulation of mammalian tooth cusp patterning by ectodin. Science 309, 2067–2070 (2005). 16179481 10.1126/science.1116848

[18] E. Järvinen, I. Salazar-Ciudad, W. Birchmeier, M. M. Taketo, J. Jernvall, I. Thesleff, Continuous tooth generation in mouse is induced by activated epithelial Wnt/beta-catenin signaling. Proc. Natl. Acad. Sci. U.S.A. 103, 18627–18632 (2006). 17121988 10.1073/pnas.0607289103 PMC1693713

[19] H. Vastardis, N. Karimbux, S. W. Guthua, J. G. Seidman, C. E. Seidman, A human MSX1 homeodomain missense mutation causes selective tooth agenesis. Nat. Genet. 13, 417–421 (1996). 8696335 10.1038/ng0896-417

[20] M. Callea, F. Fattori, I. Yavuz, E. Bertini, A new phenotypic variant in cleidocranial dysplasia (CCD) associated with mutation c.391C>T of the RUNX2 gene. BMJ Case Rep. 2012, bcr1220115422 (2012). 10.1136/bcr-12-2011-5422 PMC4542990 23220435

[21] P. Nieminen, S. Arte, D. Tanner, L. Paulin, S. Alaluusua, I. Thesleff, S. Pirinen, Identification of a nonsense mutation in the PAX9 gene in molar oligodontia. Eur. J. Hum. Genet. 9, 743–746 (2001). 11781684 10.1038/sj.ejhg.5200715

[22] I. Satokata, R. Maas, Msx1 deficient mice exhibit cleft palate and abnormalities of craniofacial and tooth development. Nat. Genet. 6, 348–356 (1994). 7914451 10.1038/ng0494-348

[23] T. Boran, H. Lesot, M. Peterka, R. Peterkova, Increased apoptosis during morphogenesis of the lower cheek teeth in Tabby/EDA mice. J. Dent. Res. 84, 228–233 (2005). 15723861 10.1177/154405910508400304

[24] R. N. D’Souza, T. Aberg, J. Gaikwad, A. Cavender, M. Owen, G. Karsenty, I. Thesleff, Cbfa1 is required for epithelial-mesenchymal interactions regulating tooth development in mice. Development 126, 2911–2920 (1999). 10357935 10.1242/dev.126.13.2911

[25] Y. Togo, K. Takahashi, K. Saito, H. Kiso, H. Tsukamoto, B. Huang, M. Yanagita, M. Sugai, H. Harada, T. Komori, A. Shimizu, M. MacDougall, K. Bessho, Antagonistic functions of USAG-1 and RUNX2 during tooth development. PLOS ONE 11, e0161067 (2016). 27518316 10.1371/journal.pone.0161067 PMC4982599

[26] C. Forscher, M. Mita, R. Figlin, Targeted therapy for sarcomas. Biol. Theory 8, 91–105 (2014). 10.2147/BTT.S26555 PMC3962319 24669185

[27] A. Mócsai, L. Kovács, P. Gergely, What is the future of targeted therapy in rheumatology: Biologics or small molecules? BMC Med. 12, 43 (2014). 24620738 10.1186/1741-7015-12-43 PMC3975154

[28] S. E. Weidauer, P. Schmieder, M. Beerbaum, W. Schmitz, H. Oschkinat, T. D. Mueller, NMR structure of the Wnt modulator protein Sclerostin. Biochem. Biophys. Res. Commun. 380, 160–165 (2009). 19166819 10.1016/j.bbrc.2009.01.062

[29] K. B. Lintern, S. Guidato, A. Rowe, J. W. Saldanha, N. Itasaki, Characterization of wise protein and its molecular mechanism to interact with both Wnt and BMP signals. J. Biol. Chem. 284, 23159–23168 (2009). 19553665 10.1074/jbc.M109.025478 PMC2755721

[30] E. Bourhis, W. Wang, C. Tam, J. Hwang, Y. Zhang, D. Spittler, O. W. Huang, Y. Gong, A. Estevez, I. Zilberleyb, L. Rouge, C. Chiu, Y. Wu, M. Costa, R. N. Hannoush, Y. Franke, A. G. Cochran, Wnt antagonists bind through a short peptide to the first β-propeller domain of LRP5/6. Structure 19, 1433–1442 (2011). 21944579 10.1016/j.str.2011.07.005

[31] K. Matoba, E. Mihara, K. Tamura-Kawakami, N. Miyazaki, S. Maeda, H. Hirai, S. Thompson, K. Iwasaki, J. Takagi, Conformational freedom of the LRP6 ectodomain is regulated by n-glycosylation and the binding of the Wnt antagonist Dkk1. Cell Rep. 18, 32–40 (2017). 28052259 10.1016/j.celrep.2016.12.017

[32] H. Kiso, K. Takahashi, S. Mishima, A. Murashima-Suginami, A. Kakeno, T. Yamazaki, K. Asai, Y. Tokita, R. Uozumi, M. Sugai, H. Harada, B. Huang, M. MacDougall, K. Bessho, Third dentition is the main cause of premolar supernumerary tooth formation. J. Dent. Res. 98, 968–974 (2019). 31238019 10.1177/0022034519858282

[33] C. Li, Y. Lan, R. Krumlauf, R. Jiang, Modulating Wnt signaling rescues palate morphogenesis in *Pax9* mutant mice. J. Dent. Res. 96, 1273–1281 (2017). 28692808 10.1177/0022034517719865 PMC5613879

[34] S. Jia, J. Zhou, C. Fanelli, Y. Wee, J. Bonds, P. Schneider, G. Mues, R. N. D’Souza, Small-molecule Wnt agonists correct cleft palates in *Pax9* mutant mice *in utero*. Development 144, 3819–3828 (2017). 28893947 10.1242/dev.157750 PMC5675451

[35] M. Pummila, I. Fliniaux, R. Jaatinen, M. J. James, J. Laurikkala, P. Schneider, I. Thesleff, M. L. Mikkola, Ectodysplasin has a dual role in ectodermal organogenesis: Inhibition of Bmp activity and induction of Shh expression. Development 134, 117–125 (2007). 17164417 10.1242/dev.02708

[36] Y. Zhang, P. Tomann, T. Andl, N. M. Gallant, J. Huelsken, B. Jerchow, W. Birchmeier, R. Paus, S. Piccolo, M. L. Mikkola, E. E. Morrisey, P. A. Overbeek, C. Scheidereit, S. E. Millar, R. Schmidt-Ullrich, Reciprocal requirements for EDA/EDAR/NF-κB and Wnt/β-catenin signaling pathways in hair follicle induction. Dev. Cell 17, 49–61 (2009). 19619491 10.1016/j.devcel.2009.05.011 PMC2859042

[37] J. T. Wright, M. Fete, H. Schneider, M. Zinser, M. I. Koster, A. J. Clarke, S. Hadj-Rabia, G. Tadini, N. Pagnan, A. F. Visinoni, B. Bergendal, B. Abbott, T. Fete, C. Stanford, C. Butcher, R. N. D’Souza, V. P. Sybert, M. I. Morasso, Ectodermal dysplasias: Classification and organization by phenotype, genotype and molecular pathway. Am. J. Med. Genet. A 179, 442–447 (2019). 30703280 10.1002/ajmg.a.61045 PMC6421567

[38] C. Kowalczyk-Quintas, L. Willen, A. T. Dang, H. Sarrasin, A. Tardivel, K. Hermes, H. Schneider, O. Gaide, O. Donzé, N. Kirby, D. J. Headon, P. Schneider, Generation and characterization of function-blocking anti-ectodysplasin A (EDA) monoclonal antibodies that induce ectodermal dysplasia. J. Biol. Chem. 289, 4273–4285 (2014). 24391090 10.1074/jbc.M113.535740 PMC3924290

[39] A. Ohazama, S. A. C. Modino, I. Miletich, P. T. Sharpe, Stem-cell-based tissue engineering of murine teeth. J. Dent. Res. 83, 518–522 (2004). 15218039 10.1177/154405910408300702

[40] K. Nakao, R. Morita, Y. Saji, K. Ishida, Y. Tomita, M. Ogawa, M. Saitoh, Y. Tomooka, T. Tsuji, The development of a bioengineered organ germ method. Nat. Methods 4, 227–230 (2007). 17322892 10.1038/nmeth1012

[41] H. Sui, A. K. Olivier, J. A. Klesney-Tait, L. Brooks, S. R. Tyler, X. Sun, A. Skopec, J. Kline, P. G. Sanchez, D. K. Meyerholz, N. Zavazava, M. Iannettoni, J. F. Engelhardt, K. R. Parekh, Ferret lung transplant: An orthotopic model of obliterative bronchiolitis. Am. J. Transplant. 13, 467–473 (2013). 23205765 10.1111/j.1600-6143.2012.04337.x PMC3638989

[42] S. Tabata, Y. Kitago, Y. Fujii, E. Mihara, K. Tamura-Kawakami, N. Norioka, K. Takahashi, M. K. Kaneko, Y. Kato, J. Takagi, An anti-peptide monoclonal antibody recognizing the tobacco etch virus protease-cleavage sequence and its application to a tandem tagging system. Protein Expr. Purif. 147, 94–99 (2018). 29550370 10.1016/j.pep.2018.03.004

[43] M. Tada, T. Suzuki, A. Ishii-Watabe, Development and characterization of an anti-rituximab monoclonal antibody panel. MAbs 10, 370–379 (2018). 29309213 10.1080/19420862.2018.1424610 PMC5916554

[44] H. Hirai, K. Matoba, E. Mihara, T. Arimori, J. Takagi, Crystal structure of a mammalian Wnt-frizzled complex. Nat. Struct. Mol. Biol. 26, 372–379 (2019). 31036956 10.1038/s41594-019-0216-z

